# Bio-inspired sensitive and reversible mechanochromisms via strain-dependent cracks and folds

**DOI:** 10.1038/ncomms11802

**Published:** 2016-07-08

**Authors:** Songshan Zeng, Dianyun Zhang, Wenhan Huang, Zhaofeng Wang, Stephan G. Freire, Xiaoyuan Yu, Andrew T. Smith, Emily Y. Huang, Helen Nguon, Luyi Sun

**Affiliations:** 1 Department of Chemical and Biomolecular Engineering and Polymer Program, Institute of Materials Science, University of Connecticut, Storrs, Connecticut 06269, USA; 2 Department of Mechanical Engineering, University of Connecticut, Storrs, Connecticut 06269, USA; 3 School of Mechanical and Electrical Engineering, Heyuan Polytechnic, Heyuan, Guangdong 517000, China; 4 Institute of Biomaterials, College of Materials and Energy, South China Agricultural University, Guangzhou, Guangdong 510642, China

## Abstract

A number of marine organisms use muscle-controlled surface structures to achieve rapid changes in colour and transparency with outstanding reversibility. Inspired by these display tactics, we develop analogous deformation-controlled surface-engineering approaches via strain-dependent cracks and folds to realize the following four mechanochromic devices: (1) transparency change mechanochromism (TCM), (2) luminescent mechanochromism (LM), (3) colour alteration mechanochromism (CAM) and (4) encryption mechanochromism (EM). These devices are based on a simple bilayer system that exhibits a broad range of mechanochromic behaviours with high sensitivity and reversibility. The TCM device can reversibly switch between transparent and opaque states. The LM can emit intensive fluorescence as stretched with very high strain sensitivity. The CAM can turn fluorescence from green to yellow to orange as stretched within 20% strain. The EM device can reversibly reveal and conceal any desirable patterns.

## Abstract (web summary)

Muscle-controlled changes in surface structures are employed in nature to achieve rapid, reversible changes in colour and transparency. Here the authors develop a simple, bilayer platform capable of several distinct analogous mechanochromic behaviours.

## Graphical abstract

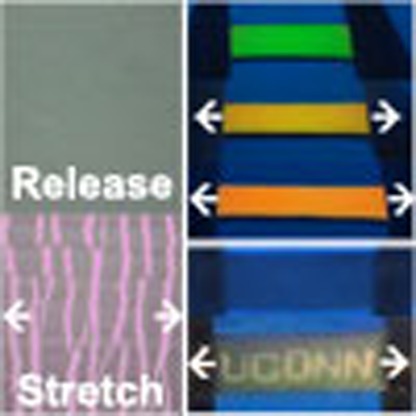

Mechanochromic devices have remarkable capabilities to change transparency and/or colour in response to mechanical stimuli, making them attractive for a wide range of applications in smart windows^1^,^2^,^3^, strain sensors^4^,^5^, encryption^2^,^6^, tunable wetting systems^3^ and so on^7^,^8^,^9^,^10^. In fact, some marine life forms have evolved camouflage traits involving dynamic and reversible alteration of their transparency^11^, fluorescence^12^ and colouration^13^,^14^,^15^ via muscle-controlled surface structures and morphologies. For example, *Hippopodius* and *Vogtia* can rapidly change from a transparent state to an opaque state when disturbed. Tactile stimulation can instantaneously evoke the contraction of radial muscles in the margin of a nectosac, resulting in a crumpled morphology with inward folds (similar to what is shown in Figs 1d and 2a), effectively reducing the transparency of the organisms^11^,^16^. Cephalopods (squid, cuttlefish, octopus and so on) are able to instantaneously change their skin colour pattern for camouflage and communication^14^,^17^,^18^,^19^. This transformation is controlled by the motion of a chromatophore, in which the sac contains pigments with radial muscles attached peripherally (Fig. 3a). In addition, the structural reflectors (for example, iridophores, leucophores, and so on.) underneath the chromatophore can reflect light from the pigment to enhance its visibility^20^. When the radial muscle is relaxed, the chromatophore has a small exposure area, on the scale of the tenth of a millimetre^14^,^19^,^21^, almost invisible to the naked eye. However, the exposure area can be rapidly expanded into the millimetre scale as the muscle contracts^22^, and the colour pattern can become instantaneously visible. The aforementioned two organisms demonstrate different optical properties by altering the surface structures and morphologies of their muscles via mechanical means. Inspired by this display strategy found in nature, we pursue here deformation-controlled surface engineering to achieve various mechanochromisms through a series of optical device designs. The key element is to develop a hybrid bilayer material system consisting of a rigid thin film bonded on a soft substrate to accomplish four different types of mechanochromisms: (1) transparency change mechanochromism (TCM), (2) luminescent mechanochromism (LM), (3) colour alteration mechanochromism (CAM) and (4) encryption mechanochromism (EM). All of these device types can rapidly and reversibly change their appearance when subjected to small mechanical stimuli (<40% uniaxial tensile strain; all discussions of strain in this paper are restricted to uniaxial tensile strain). For example, the TCM device can reversibly switch between transparent and opaque states. The LM can emit intensive fluorescence as stretched with an ultrahigh strain sensitivity as compared with the electrical resistance-based strain sensor. The CAM can turn fluorescence from green to yellow to orange as stretched within 20% strain. The EM device can reversibly reveal and conceal any desirable patterns within 17% strain. The success of the designs lies in controlling the surface structures and morphologies of these devices during the deformation, that is, the evolution of the cracks and invaginated folds in the top thin film.

## Results

### Design and preparation of the mechanochromisms

**Figure 1:**
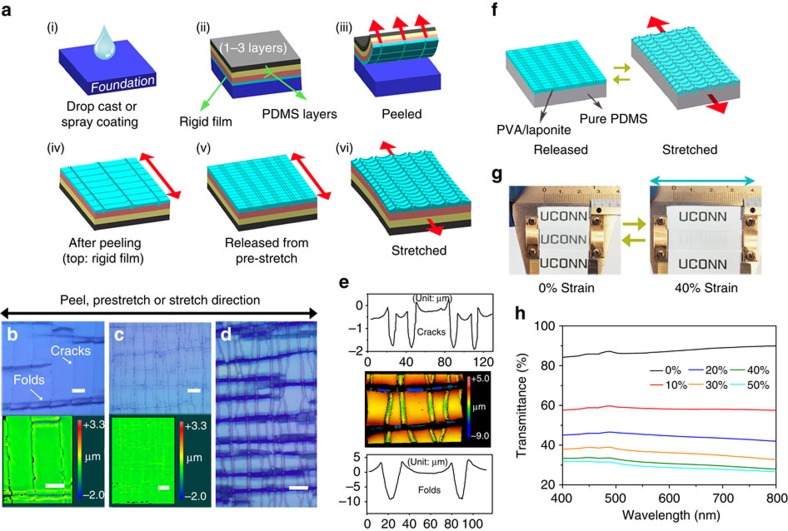
Preparation of mechanochromisms and strain responsive properties of TCM. (**a**) General preparation approach for all the mechanochromisms (the red arrows indicate the peel, pre-stretch or stretch direction); (**b**–**e**) optical microscope images and surface profiles of the topmost rigid layer of the TCM; (**b**) immediately after being peeled from the foundation (corresponding to step (iv)); (**c**) release from 60% strain pre-stretch (corresponding to step (v)), (**d**,**e**) stretched at 40% strain (corresponding to step (vi)); (**f**) design scheme for the TCM; (**g**) digital photos demonstrating the TCM; (**h**) strain-dependent transmittance of the TCM. Scale bars, 20 μm. Objects in the schematic diagram are not drawn to scale.

**Figure 2:**
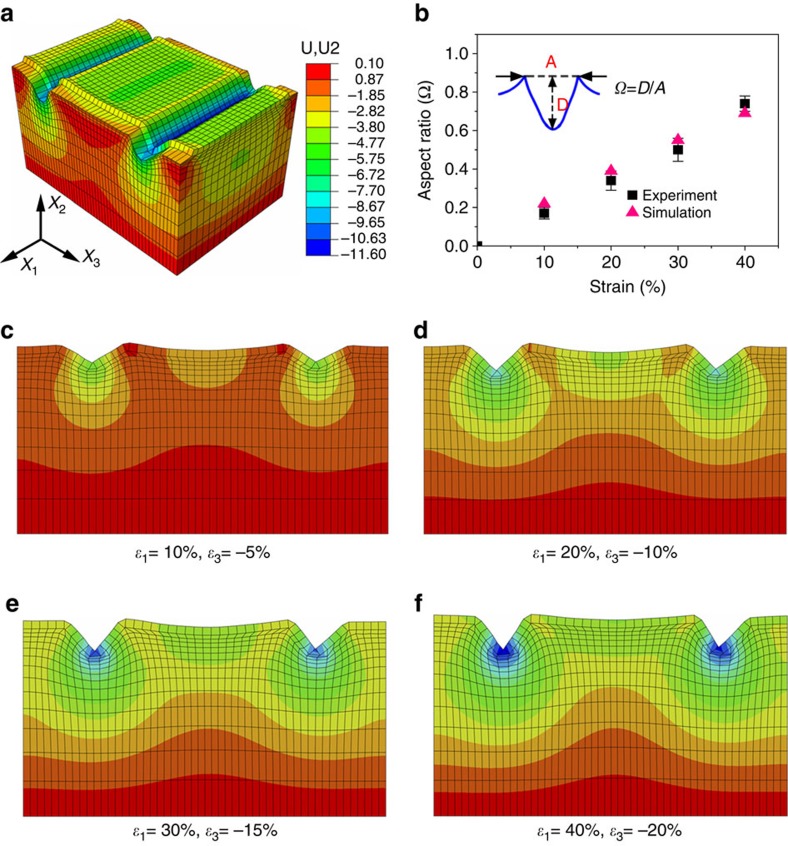
Simulated or experimental results of folds in the TCM. (**a**) 3D finite element simulation of folding as subjected to 40% tensile strain in the X_1_ direction, accompanied by 20% compression in the X_3_ direction due to the Poisson effect (unit: μm); (**b**) experimental and simulated results of the folding's aspect ratio with different applied tensile strains; error bars are defined as s.d.; (**c**–**f**) simulated evolution of folding (visualized as two-dimensional cross-section in the X_1_ direction), with increasing tensile strain in the X_1_ direction. All figures use the same stress scale bar as applied in **a**.

**Figure 3:**
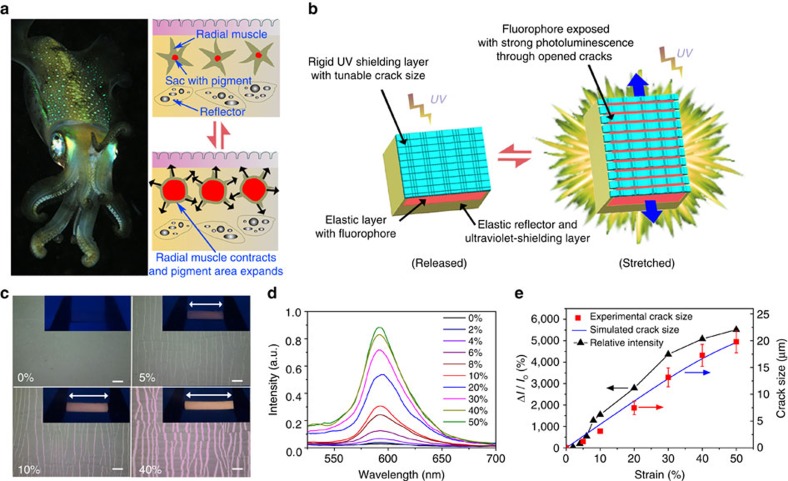
Design strategy and mechanical switchable fluorescence of the LM. (**a**) (Left) cephalopods (squid) showing chromatophore on the skin (photo courtesy of Nhobgood (Own work; CC BY-SA 3.0)) and (right) the colouration mechanism adopted by cephalopods; (**b**) design scheme of the reversible LM; (**c**) optical microscope images showing the distribution and size of the longitudinal cracks upon strain in the LM (scale bars, 100 μm); the insets are digital photos of this device experiencing corresponding strains under ultraviolet light (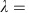
365 nm; white arrow indicating stretch direction); (**d**) fluorescent spectra of the LM as a function of strain (excitation wavelength of ultraviolet=365 nm); (**e**) relative fluorescence intensity ratio at each strain to released state (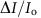
=intensity at given strain/intensity at 0% strain—1) and the experimental and simulated cracks size evolution with strain for LM. Error bars are defined as s.d.

**Figure 4:**
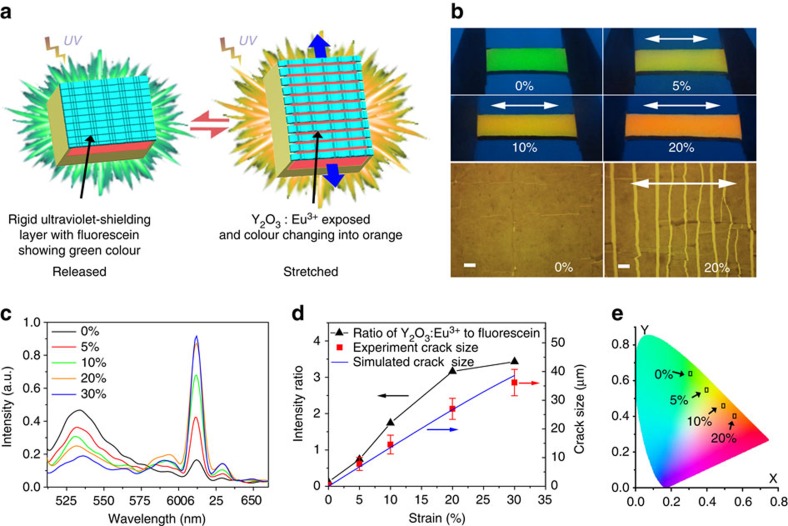
Design strategy and mechanical responsive colour change of the CAM. (**a**) Design scheme of the reversible CAM; (**b**) digital photos of CAM at different strains (0–20%) under ultraviolet light (*λ*=254 nm), and the corresponding optical microscope images of crack size and distribution at 0 and 20% tensile strain (white arrow indicating stretch direction); (**c**) fluorescent spectra of the CAM as a function of strain; (excitation wavelength of ultraviolet=247 nm); (**d**) the change of intensity ratio of Y_2_O_3_:Eu^3+^ to fluorescein with strain in the CAM and the corresponding experimental and simulated crack size evolution with strain; error bars are defined as s.d.; (**e**) the colour change of CAM at different strains illustrated in the CIE colour space.

**Figure 5:**
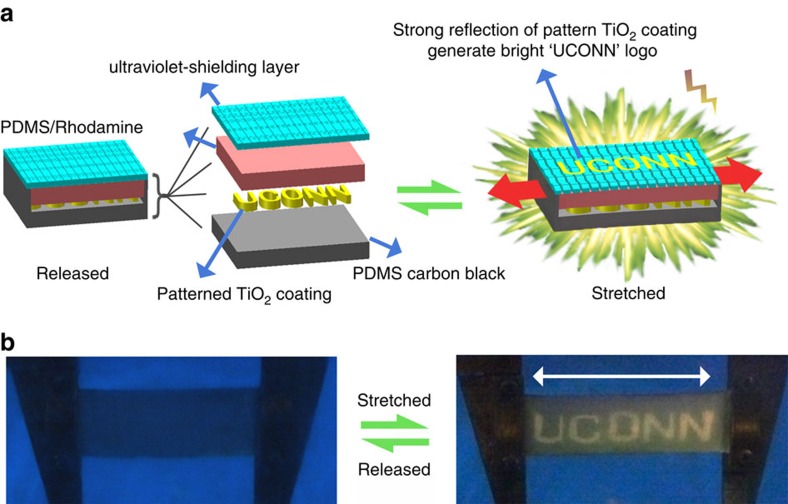
Design strategy and mechanical responsive encryption properties of the EM. (**a**) Design scheme of the reversible EM; (**b**) the hidden ‘UCONN' logo concealed at released state and revealed upon being stretched to 17% strain with excellent reversibility. (Excitation wavelength of ultraviolet=365 nm).

To obtain a sensitive and reversible TCM, the device is designed to contain a transparent rigid film (made of polyvinyl alcohol (PVA)/laponite composite) tightly bonded to a soft polydimethylsiloxane (PDMS), as shown in Fig. 1f. The device can reversibly exhibit conspicuous visual change between a transparent state and an opaque state upon stretching and releasing within 40% strain (Fig. 1g and Supplementary Movie 1). The opacity of the stretched state can be attributed to strong trapping and scattering of light resulting from the strain-dependent cracks and folds (Figs 1d and 2a), in analogy to surface structures in *Vogtia* resulting in an opaque state. It should be noted that this device can be fabricated using a facile and scalable method without using specialized equipment utilizing plasma processing^2^, ultraviolet-ozone radiation^3^, or lithography^1^. For the LM, a rigid ultraviolet-shielding film is firmly adhered to a soft PDMS substrate that contains a fluorophore layer and a reflector layer (Fig. 3b). When the device undergoes stretching, distributed cracks develop in the ultraviolet-shielding layer, with crack size correlated to the applied tensile strain. These cracks act as ‘gates' to adjust the exposure area of the fluorophore and the concomitant ultraviolet-excited fluorescent intensity. The additional reflector layer at the bottom can enhance the fluorophore luminescence via reflection, leading to a significant improvement of the strain-responsive luminescent performance. Only 5% strain is sufficient to change the visibility of the device from a nonluminous state to an apparently eye-detectable luminescent state (see Supplementary Movie 2). The design strategy used for the LM, including the surface cracks and the reflector layer, is inspired by the structure of the chromatophores in cephalopods. The structure of the CAM is similar to the LM, but in this design, a thin film of laponite/fluorescein with green fluorescence is coated atop the rigid ultraviolet-shielding film, and the rigid layer is subsequently adhered to a soft layer that contained europium-doped yttrium oxide (Y_2_O: Eu^3+^, emitting orange fluorescence; Fig. 4a). The device exhibits green fluorescence in the released state with all the cracks closed. At only 20% strain, crack opening in the rigid film can significantly increase the exposure area of PDMS/Y_2_O_3_:Eu^3+^ layer, generating orange fluorescence. Thus, fluorescence from the device changes in colour from green to yellow to orange with increasing strain (Fig. 4b and Supplementary Movie 3). In other research, the CAM or the LM is commonly realized by incorporating special synthetic dyes into a polymer matrix^5^,^23^,^24^,^25^,^26^,^27^. Mechanical strain can alter the molecular configurations of the dye molecules and thus change the resulting fluorescent colours^26^,^27^,^28^,^29^. However, these dyes require special synthesis expertise and demanding reaction conditions, and the threshold strain to generate conspicuous visual change is generally large (100–500%). Their insensitivity to smaller strains (0–40%) has limited potential applications in strain-sensing^5^,^25^,^26^,^27^, while our devices demonstrate high sensitivity in this strain range achieved with facile fabrication processes and commercially available dyes. For the EM, the device is modified based on the LM with the encrypted information embedded in the soft PDMS layer, as shown in Fig. 5b. As a result, the information ‘input' to the device can be reversibly revealed and concealed upon stretching and releasing the sample (0–17% strains) under ultraviolet light (Fig. 5b and Supplementary Movie 4).

Preparation of the aforementioned mechanochromisms follows generally similar paths, as illustrated in Fig. 1a. Initially, a thin rigid composite film (could contain multilayers) was prepared by drop-casting or spray-coating on a plastic foundation followed by the treatment of vinyl-functionalized silane vapour (step (i)). A liquid PDMS substrate was then cast atop the thin film and then thermally cured to form a thick, soft layer (*ca.* 1 mm). This process can be repeated to cast multiple PDMS layers containing different functional components (step (ii)). The approach allows the low surface energy liquid PDMS (20.4 mN m^−N^)^20^ to form an intimate contact and strong adhesion with the rigid thin film via covalent bonding^28^,^29^. The device was then carefully peeled away from the foundation along one direction (step (iii)). After peeling, the topography of the rigid film exhibited periodical longitudinal cracks vertical to the peeling direction and transverse folds perpendicular to the cracks because of the compressive force as a result of the Poisson effect (step (iv))^30^,^31^,^32^,^33^. However, this rough surface would deteriorate the transparency at the released state for the TCM, and the low crack density would also limit the strain-dependent performance for all the mechanochromisms. Thus, a pre-stretch of 60% strain was applied to increase the densities of the cracks and the folds in the rigid thin film. It should be noted that such a pre-stretch results in damage at the edges and valleys of the folds. Upon release, the rigid film restored to a flattening surface with dense cracks fully closed (step (v)). The typical optical microscope images and surface profiles at the aforementioned procedures are shown from Fig. 1b–e. When the device was stretched again, the rigid thin film showed a quasi-orthogonal periodic network with distributed longitudinal cracks and transverse invaginated folds (step (vi) and Fig. 1d–e) because of the mismatch of stiffness between the rigid thin film and the soft PDMS substrate, and the strong interface bonding in between^34^,^35^,^36^. All of these devices exhibited reversible and durable performance, and could be cycled extensively with little change in response characteristics between step (v) and step (vi) within the elasticity range of PDMS (see Supplementary Figs 1–4).

### Strain-dependent optical properties of TCM

The correlation between the optical transmittance and the applied strain for the TCM is demonstrated in Fig. 1h. The sample exhibited high transparency (transmittance >88% at 600 nm) in the released state (Fig. 1g). With applied strains, the transmittance dropped sharply by nearly half in the first 20% strain because of light scattering and trapping by the invaginated folds and longitudinal cracks at the microscale^37^. The sample became highly opaque (transmittance <29% at 600 nm; Fig. 1h) when being stretched to 40% strain. This behaviour persists over a very large number of cycles (>50,000 times) without significant change in the optical properties (Supplementary Fig. 1), making them promising for applications in smart windows and dynamic optical switches^2^. As shown in the surface profile image (Fig. 1e), the opening size of the longitudinal cracks was increased up to *ca.* 7.9 μm with an average depth of *ca.* 1.7 μm as stretched to 40% strain. Meanwhile, the crest-to-bottom depth of the invaginated folds increased sharply to 13.3 μm with a crest-to-crest distance of 17.8 μm. These folds show a unique feature of sharp double ridges (or edges), indicating that the ridges undergo out-of-surface traction, while the valley experiences into-surface traction. These folds show a high aspect ratio (Ω) of 0.75 (defined as the ratio between the crest-to-bottom depth (D) to crest-to-crest distance (A)) when subjected to 40% strain, which accounts for the significantly enhanced light-trapping capability and the surface hydrophobicity^14^,^37^. As a result, the device can change between transparent and opaque states in response to small strains (0–40% strain). This unique fold–ridge formation mechanism was simulated through a three-dimensional (3D) finite element (FE) model using the commercially available software, ABAQUS (version 6.14), as shown in Fig. 2a. The rigid thin film was tied on the PDMS substrate by enforcing the displacement continuity at the interface. Modelling details, including the geometry, boundary conditions and material properties, are given in the Supplementary Methods and Supplementary Fig. 5. The key to capture the reversible fold–ridge formation is to introduce damage in the thin film, which results from the development of invaginated folds during the pre-stretch stage. These softening areas, modelled as a damaged solid by reducing the Young's modulus to 1% of the modulus of the pristine thin film, promote the formation of high-aspect-ratio folds in the stretched state, resulting in an opaque state due to the light-trapping capability of these folds. Figure 2c–f shows the evolution of the folding geometry (cross-section cut along the tensile direction) with increasing strains obtained from the FE simulation. The aspect ratio computed using the FE model is in good agreement with the experiment, as shown in Fig. 2b. It is worth mentioning that the surface wettability of the TCM can also be tuned by elongation strain (see Supplementary Fig. 6), which is valuable for many applications, including those in liquid transportation^38^,^39^, smart microfluidics devices^40^,^41^ and integrated surfaces and devices^42^,^43^.

### Mechanically responsive fluorescence of the LM

Since TiO_2_ has a high refractive index (2.61 at 600 nm)^44^ and an excellent ability to block ultraviolet light through absorption, scattering and reflection^45^, a TiO_2_/PVA (mass ratio=4:1) composite was utilized as an ultraviolet-shielding layer in the LM (Fig. 3b). The soft PDMS substrate contains a Rhodamine-filled luminescent layer and a TiO_2_-filled reflector-reflective layer, as shown in Fig. 3b. The structural reflector can not only shield the ultraviolet light from other angles but also significantly improve the overall fluorescence intensity by reflecting the luminescence from fluorophore. Figure 3(c) shows that the device has no eye-detectable fluorescence at the released state with cracks closed in the rigid thin film. When stretched to 5% strain, the device can display conspicuous luminescence with the presence of the open cracks that are shown as bright strips in the background under an optical microscope (Fig. 3c), indicating the remarkable strain-responsive sensitivity of this device. The fluorescence spectra (Fig. 3d) and relative intensity ratio (RIR; defined as (intensity/intensity at 0% strain−1), Fig. 3e) as a function of strain quantitatively demonstrate the variation of strain-dependent luminescence. Impressively, when stretched to 40% strain, the device displays a strong luminescence increased by 55 times in RIR (Fig. 3e) with a crack-opening size of 18 μm. For electrical resistance strain sensors, their gauge factor is defined as (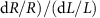, where *R* is the resistance and *L* is the length. The gauge factor for a conventional metal sensor is *ca.* 2.0 and the value for a polymer composite (such as PDMS/carbon black) is *ca.* 20 (refs 46, 47, 48). Similarly, we define the slope (=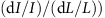 of the RIR curve in Fig. 3e as the gauge factor for our samples. The gauge factor of our device is 123.7 (0–50% strain), which is significantly higher than many reported gauge factors in the electrical resistance strain sensors^46^,^47^,^48^,^49^,^50^. The sensitivity of the strain-responsive luminescence in the LM can be attributed to the strain-induced opening of the originally closed cracks, a high crack density with small intercracks spacing (spacing size=*ca*. 41 μm), and the strong reflection from the structural reflector. The evolution of RIR can be correlated to the exposure area of fluorophore, which is dictated by the crack-opening size. Thus, both the crack-opening size and RIR are evolved in a similar trend. Here these opening cracks act as microscale ‘gates' that allow the exposure of fluorophore to the ultraviolet light and ‘switch on' the corresponding fluorescence. To demonstrate the importance of the TiO_2_-filled reflective layer, we prepared a LM device with a PDMS/carbon black reflector. It was found that the RIR of this device was *ca.* 1.2 at 40% strain, which is ∼46 times smaller than the one using the PDMS/TiO_2_ reflector. Thus, the strong reflective luminescence of the fluorophore from the reflector is another key factor to enhance the strain-responsive fluorescence.

The evolution of the crack size in the LM was simulated using a 3D FE model similar to that for the fold–ridge formation in the TCM, in which the displacement continuity was enforced across the interface between the rigid thin film and the PDMS substrate. The distributed cracks in the thin film, which were developed after the pre-stretch stage, were modelled as dummy nodes embedded at the crack interface. These cracks were fully opened across the thin film and arrested in the PDMS substrate, as schematically shown in Supplementary Fig. 7. The simulation captures the nonlinear crack-opening response for strains greater than 30%, which are in good agreement with the experimental results, as shown in Fig. 3e. In the small-strain regime (*ɛ*<20%), the computed crack size grows linearly with increased strains, while the experiment shows a nonlinear increase of crack size and RIR. The computed crack size is slightly larger than the experimental result because of the fact that the model neglects the cohesive force at the crack interface. The strong intermolecular hydrogen bonding in the thin film (PVA/TiO_2_ layer) allows the crack to be fully closed at the released state^51^. When the film–substrate system is subjected to small strains, there exist cohesive forces that reduce the crack-opening size, resulting in an exponential growth of the RIR in the range of 0–8% strain (Supplementary Fig. 8 and Supplementary Fig. 9). However, the effect of cohesion disappears once the crack is widely open with the fracture energy completely dissipated.

### Strain responsive properties of the CAM and the EM

The CAM is achieved by coating a laponite/fluorescein film with green fluorescence atop the TiO_2_/PVA thin film, which is bonded to a PDMS layer containing Y_2_O_3_:Eu^3+^ with orange fluorescence (Fig. 4a). The overall fluorescent colour is determined by the linear combination of green and orange fluorescence. To achieve a conspicuous colour alternation in small strains less than 20%, it is necessary to consider the ratio of the exposure areas of the two fluorophores, which equals to the area ratio of opening cracks (corresponding to Y_2_O_3_:Eu^3+^) and non-opening-crack surface (corresponding to fluorescein). For example, at 20% strain, this ratio can be calculated from the area of bright strips (corresponding to opening cracks) divided by the area of dark background (corresponding to non-opening-crack surface) in an optical microscope image shown in Fig. 4b. This ratio (Supplementary Fig. 10) is *ca.* 19 at 20% strain, suggesting that the fluorescent intensity and the concentration of Y_2_O_3_:Eu^3+^ need to be much higher than those of fluorescein to compensate the small exposure area. Thus, the concentrations of fluorescein and Y_2_O_3_:Eu^3+^ were optimized to be 1 × 10^−8^ and 4.3 × 10^−5^ mol g^−1^ in their matrix, respectively, to maximize the strain-dependent colour alternation performance. The fluorescent spectra (excited at 247 nm) as a function of strain (Fig. 4c) show two main peaks at 533 nm (from Fluorescein) and 612 nm (from the ^5^D_0_→^7^F_2_ transition of Eu^3+^ in Y_2_O_3_:Eu^3+^)^52^. The peak intensity at 533 nm gradually decreased with an increasing strain, while the peak intensity at 612 nm increased significantly. Notably, when the strain was increased to 5% or higher, new small peaks at 594 nm (^5^D_0_→^7^F_1_ transition of Eu^3+^) and 629 nm (^5^D_0_→^7^F_2_ transition of Eu^3+^) appeared owing to the fluorescence from Y_2_O_3_:Eu^3+^ (refs 53, 54). Extrapolated from the spectra, the intensity ratio of Y_2_O_3_:Eu^3+^ to fluorescein generally increases in a nonlinear manner with an increasing strain (especially in the range of 20–30% strain), similar to the trend of crack-opening size, as shown both in the experiment and simulation (Fig. 4d). The intensity ratio of Y_2_O_3_:Eu^3+^ to fluorescein is greater than 1 at 10% strain, and the ratio further increases to *ca.* 3.5 at 20% strain (Fig. 4d). Here the applied strains resemble a palette that can tune the fluorescent colour of the device from green to yellow to orange within 20% strain (Fig. 4b). The FE model for computing the crack-opening size (see Supplementary Methods for modelling detail) is similar to that for the LM; however, different values of crack-spacing and depth were used in the simulation, as summarized in Supplementary Table 1. The computed crack size is in good agreement with the experiment, as shown in Fig. 4d. The model is able to capture the nonlinear increase in the crack-opening size between 20 and 30% strains, which agrees with the experimental results of the intensity ratio and the crack size in the same strain range. The colour coordinates in different strains are defined by the Commission Internationale de L'Eclairage (CIE) colour space coordinate calculated from the strain-dependent fluorescent spectra. As shown in Fig. 4e, the coordinates change linearly from green to yellow to orange with an increasing strain. Since the CIE coordinate is a linear combination of individual colour coordinates, each coordinate represents a colour combination of two individual fluorescence, one from fluorescein and the other from Y_2_O_3_:Eu^3+^. Only 20% strain is necessary to lead the device to reach orange (coordinate=0.55, 0.41) from green (0.30, 0.63) in original released state with strong colour alternation and large colour space crossing. In addition, in this device, the transition between any two visible colours in the CIE coordinate other than green to orange can be achieved by simply incorporating the other corresponding fluorophores. Thus, we have introduced a facile and universal method to prepare a sensitive and reversible mechanochromic device, which can offer strain-sensitive colour-alternation signal to potentially visualize the occurrence of mechanical failure.

Furthermore, the design of EM, which can be applied in encryption or display optics are shown in Fig. 5a. Below the ultraviolet-shielding layer and PDMS/Rhodamine, a patterned TiO_2_ coating with ‘UCONN' logo is placed atop the PDMS/carbon black layer. As discussed above, the strain-responsive sensitivity of the LM using PDMS/TiO_2_ as a reflector is much higher than that using PDMS/carbon black. Stretching this device under ultraviolet light (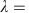
365 nm) at 17% strain allowed the area having the TiO_2_ coating as the reflector to display the ‘UCONN' logo with much stronger luminescence than the other areas, while the letters can be well concealed as released (Fig. 5b). Thus, the hidden ‘UCONN' logo can be reversibly revealed and concealed upon stretching and releasing the sample (0% -17% strains) under ultraviolet light.

## Discussion

In our device-preparation procedures, liquid PDMS was added on the thin film via drop casting. This procedure allows part of the uncured PDMS to tightly bond with the thin film, leading to a strong interface between the two layers. The strong interface is essential for achieving reversible and durable strain-dependent optical response. An alternative approach was tried by directly casting the thin film on the cured PDMS substrate, and interfacial delamination was observed immediately after the first stretching and releasing cycle. As a result of the poor interfacial adhesion, no conspicuous mechanical responsive transparency change was observed. In addition, our preparation procedure can create localized closed cracks on the rigid thin film in the peeling and pre-stretch stage. The localized damage plays an important role in directing the formation of folds in the step (vi) shown in Fig. 1a. This folding structure is generated instantaneously under a very small applied strain as shown in Supplementary Fig. 11 (3% tensile strain applied with a corresponding 1.5% compression in the vertical direction because of the Poisson's effect). This unique feature could inspire the design of folding surface on other system. In addition, the excellent light-trapping capability of the high aspect ratio folding surface in the TCM can be used to increase the efficiencies of solar energy-harvesting systems^37^.

For the LM and CAM, the opening and closing of the cracks that penetrated into the interface between the rigid thin film and PDMS substrate is the key to achieving mechanically responsive optical properties. An alternative approach has been tried via casting liquid PDMS containing Rhodamine dye on the porous pure TiO_2_ particulate film to prepare LM. Owing to the low surface tension of liquid PDMS, it can effectively penetrate into the porous spacing of the TiO_2_ network. The resulting device does not show eye-detectable fluorescence even when stretched to 40% strain. The result follows from the absence of penetrated cracks in this system as it is stretched because of the elastic nature of the PDMS-infused TiO_2_ thin film. Stretching this device can only slightly reduce the concentration of TiO_2_ per unit area, while the remaining substantial amount of TiO_2_ still effectively blocks the ultraviolet light from travelling into the PDMS/fluorophore layer. Thus, PVA was mixed with TiO_2_ particles to form an impenetrable thin film for PDMS, and the rigid nature of PVA/TiO_2_ film allows the formation of cracks on the thin film that penetrated into the interface as stretched. The ultraviolet-blocking effect can be significantly reduced with applied strain to allow the ultraviolet to travel through and excite fluorescence in the PDMS/fluorophore layer.

In summary, we have designed a series of mechanochromic devices inspired by nature with capabilities ranging from changing transparency, switchable luminescence, to altering colouration, revealing and concealing patterns in response to mechanical stimuli. The key to accomplish these optical properties is establishing strain-induced control over longitudinal crack-opening and transverse invaginated folds. All of these devices comprises a rigid thin layer atop PDMS elastomer based on highly accessible, low-cost materials compatible with facile and quick fabrication. For TCM, the folds and cracks capable of strongly trapping and scattering light can endow the originally transparent samples with opacity. The evolution of crack-opening and fold-ridge mechanisms is captured through FE analysis that incorporates damage and cracks in the rigid thin layer. For LM, the strain-tunable cracks on the ultraviolet-shield layer act as ‘gates' to modulate ultraviolet transmission to ‘switch on/off' the luminescence of mechanochromism. This device exhibits a remarkably high strain-responsive sensitivity with a gauge factor of *ca.* 123.7, which is significantly higher than some strain sensors based on electrical resistance change, demonstrating a potential for sensing mechanical failure or damage. Two devices with capabilities of colour alternation and encryption are also demonstrated in the report. All the mechanochromisms show outstanding durability and reversibility, which can preserve the strain-responsive performance on stretching and releasing for virtually infinite cycles within the elasticity range. In this work, we were inspired by the display and colouration mechanism from biological organisms, that is, using the muscle strain to control surface structure and morphology and tune the resulting optical properties. We therefore use the strain-dependent cracks and folds on the rigid thin film of a series of well-designed devices as examples to show how mechanically controlled surface engineering can achieve durable and diverse mechanochromic optical responses. We believe that these design strategies can inspire various designs of other sensitive and reversible stimulus-responsive materials with widespread applications.

## Methods

### Preparation of TCM

Transparent PVA (Mowiol 8–88, *M*_w_∼67,000 from Kuraray) and laponite (BYK Additives Inc., Gonzales, TX, USA, mass ratio=1:4) composite films with a thickness of *ca.* 1.5 μm were cast on a pre-cleaned foundation, followed by treatment with vinyltrimethoxysilane vapour for 2 h. Pure liquid PDMS (Sylgard-184, Dow Corning, base to curing agent ratio=10:1, thickness: ∼1 mm) layer was then coated on the PVA/laponite composite film and placed at room temperature for 12 h, followed by thermal curing at 80 °C for 2 h. The cured bilayer sheet was carefully peeled away from the foundation towards one direction. The peeling speed was controlled at *ca.* 10 mm s^−1^ at an angle of *ca.* 45°, and the peeling area was *ca.* 25 cm^2^. It was then cut into rectangles (*ca.* 40 mm × 10 mm) and mounted on a custom-built stretching tool. A pre-stretching of 60% uniaxial strain was applied along the initial peeling direction. The released sample was then ready for various performance tests.

### Preparation of LM

PVA (Mowiol 8–88, *M*_w_∼67,000 from Kuraray) and TiO_2_ (99.9%, CR828, Tronox; mass ratio=1:4) composite films with a thickness of *ca.* 5.1 μm were spray-coated by an airbrush style spray-gun (Master Airbrush G444-SET, needle nozzle 0.5 mm and Royal Mini Air Compressors, TC-20B, 50 mg ml^−1^ PVA/TiO_2_ aqueous suspension was used) on a pre-cleaned foundation followed by treatment with vinyltrimethoxysilane vapour for 2 h. A layer of PDMS/Rhodamine (99.9%, Alfa Aesar) composite film (Rhodamine concentration: 4.8 × 10^−5^ mol g^−1^, thickness: ∼1 mm) and a layer of PDMS/TiO_2_ composite film (mass ratio=17:1, thickness ∼1 mm) was then formed atop the TiO_2_-PVA film by repeating the aforementioned drop-casting and curing procedures. The PDMS layers contain the same concentration of curing agent (base to curing agent ratio=10:1). The rest of the process was the same as TCM preparation.

### Preparation of CAM

A layer of PVA/laponite (mass ratio=1:4) composite film containing fluorescein (>90%, Alfa Aesar, fluorescein concentration: 1 × 10^−8^ mol g^−1^) was drop-cast on a pre-cleaned foundation before the spray-coating of another layer of PVA/TiO_2_ composite film (mass ratio=1:4; total thickness of these two layers: ∼13.9 μm) using the aforementioned airbrush style spray-gun followed by treatment with vinyltrimethoxysilane vapour for 2 h. The PDMS/Y_2_O_3_:Eu^3+^ (>99%, Sigma-Aldrich, dye concentration: 4.3 × 10^−5^ mol g^−1^, thickness: ∼1 mm) and PDMS/TiO_2_ (mass ratio=17:1, thickness: ∼1 mm) layers were drop-cast atop the thin film by repeating the aforementioned drop-casting and curing procedures. The PDMS layers contain the same concentration of curing agent (base to curing agent ratio=10:1). The rest of the sample preparation is the same as the TCM.

### Preparation of EM

A layer of PVA/TiO_2_ (mass ratio=1:4, thickness: ∼5.1 μm) composite films were spray-coated on a pre-cleaned foundation by the aforementioned airbrush style spray-gun. A layer of PDMS/Rhodamine (Rhodamine concentration: 4.8 × 10^−5^ mol g^−1^, thickness: ∼1 mm) was then dropped and cured atop the PVA/TiO_2_ film. Subsequently, a thin layer of patterned TiO_2_ film was spray-coated with the assistance of a stencil mask atop the PDMS/Rhodamine layer. Finally, a layer of PDMS/carbon black (mass ratio=100:3, thickness ∼1 mm) was deposited on the top of the aforementioned multilayer structure. The rest of the sample preparation is the same as the LM.

### FE simulation

The FE simulation of crack evolution was carried out using the commercial software ABAQUS (version 6.14). The schematic FE model for the folding in TCM is shown in Supplementary Fig. 5. The geometric parameters and boundary conditions for the crack-opening response are shown in the Supplementary Table 1 and Supplementary Fig. 7, respectively. A detailed discussion about the FE simulation for these two systems is available in the Supplementary Methods.

### Characterization

The mechanochromism samples were cut into rectangle shape (*ca.* 40 mm × 10 mm) and mounted on a custom-built stretching tool to determine the optical performance. The morphology of the topmost rigid thin film with different strains under the transmission (for LM and CAM) and reflective mode (for TCM) were recorded on an optical microscope (AmScope ME 520TA). The strain-dependent surface profile of the topmost rigid thin film of the TCM was examined on a ZYGO NewView 5000 non-contact white-light profilometer. The strain-dependent transmittance test for the TCM was conducted on a Perkin Elmer ultraviolet/visible/near-infrared Lambda 900 spectrophotometer from 400 to 800 nm. Fluorescent spectra for LM and CAM were examined on a Jobin Yvon Fluorolog-3 fluorimeter with an excited light source at 365 or 247 nm. All of the digital photos and videos were captured with a Sony DSC-HX9V digital camera. All of the fluorescent samples were placed in a UVP Chromato-Vue C-70G ultraviolet viewing system with a ultraviolet light source of 365 or 254 nm for photographing or videotaping. Cyclic fatigue test of the samples was conducted on an Instron 5500 universal testing machine. Contact angle for TCM was tested on a Pendant Drop Tensiometer OCA 20 from Future Digital Scientific Corp.

## Additional information

**How to cite this article:** Zeng, S. *et al*. Bio-inspired sensitive and reversible mechanochromisms via strain-dependent cracks and folds. *Nat. Commun.* 7:11802 doi: 10.1038/ncomms11802 (2016).

## Supplementary Material

Supplementary Figures, Supplementary Table, Supplementary Methods and Supplementary References Supplementary Figures 1-11, Supplementary Table 1, Supplementary Methods and Supplementary References

Supplementary Movie 1 Transmittance Changed Mechanochromism (TCM) shows transparency variation when uniaxially stretched and released within 40% strain.

Supplementary Movie 2 Luminescent Mechanochromism (LM) shows switcheable fluorescence with uniaxial strain within 10%.

Supplementary Movie 3 Color Alternation Mechanochromism (CAM) shows color change between green and orange upon uniaxial stretching and release within 20% strain.

Supplementary Movie 4 Encryption Mechanochromism (EM) shows a "UCONN" logo can be reversibly revealed and concealed upon uniaxial stretching and release within 17% strain.
