# Supplementary material for: Bio-inspired sensitive and reversible mechanochromisms via strain-dependent cracks and folds
Source: Nat Commun. 2016 Jul 8;7:11802. doi: 10.1038/ncomms11802 (PMC4941047; doi:10.1038/ncomms11802)
Supplement: Supplementary Figures, Supplementary Table, Supplementary Methods and Supplementary References — Supplementary Figures 1-11, Supplementary Table 1, Supplementary Methods and Supplementary References [file ncomms11802-s1.pdf]

Supplementary Figures

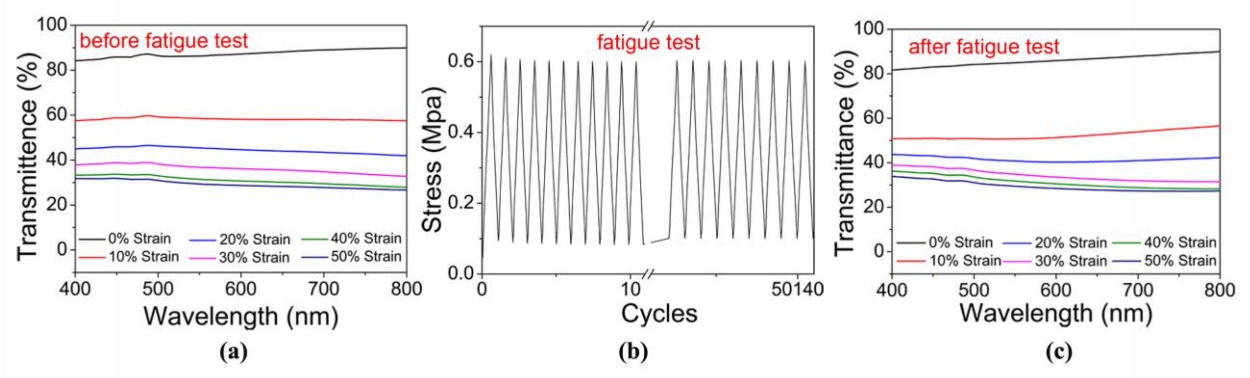

Supplementary Figure 1. (a) Transmittance spectra of the TCM at different strains as tested before the fatigue test; (b) correlation between cyclic stress and cycles curve for the fatigue test; (c) transmittance spectra of the TCM at different strains as tested after the fatigue test.

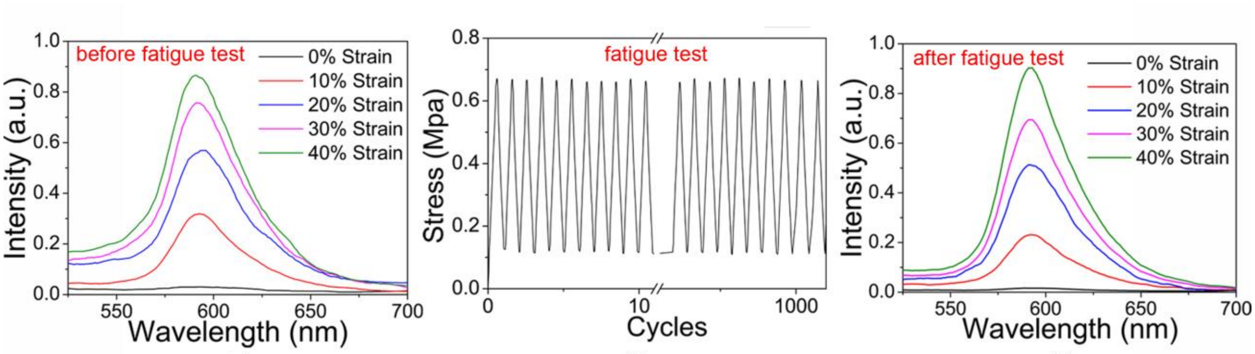

(a) **(b)** **(c)**

Supplementary Figure 2. Fluorescence spectra of the LM at different strains as tested before the fatigue test (excitation wavelength of UV = 365 nm); (b) correlation between cyclic stress and cycles curve for the fatigue test; (c) fluorescent spectra of the LM at different strains as tested after the fatigue test (excitation wavelength of UV = 365 nm).

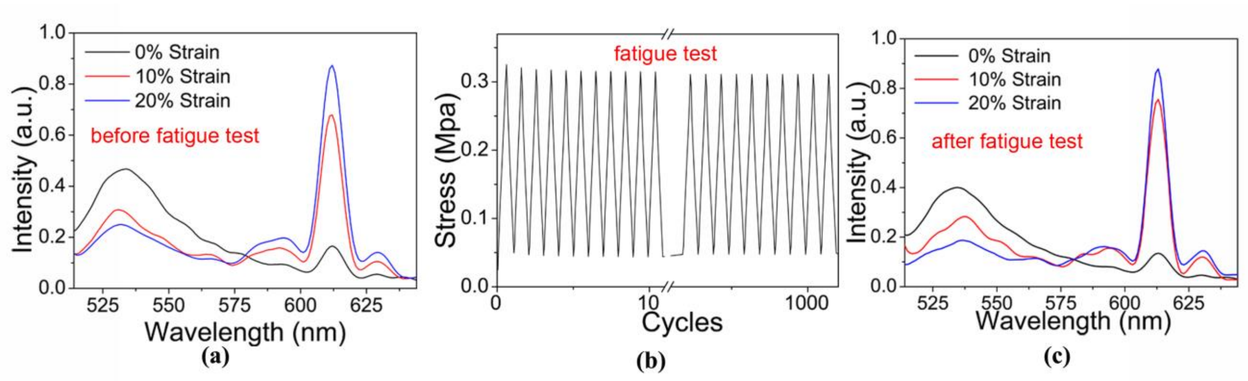

Supplementary Figure 3. (a) Fluorescence spectra of the CAM at different strains as tested before the fatigue test (excitation wavelength of UV = 247 nm); (b) correlation between cyclic stress and cycles curve for the fatigue test; (c) fluorescence spectra of the CAM at different strains as tested after the fatigue test (excitation wavelength of UV = 247 nm).

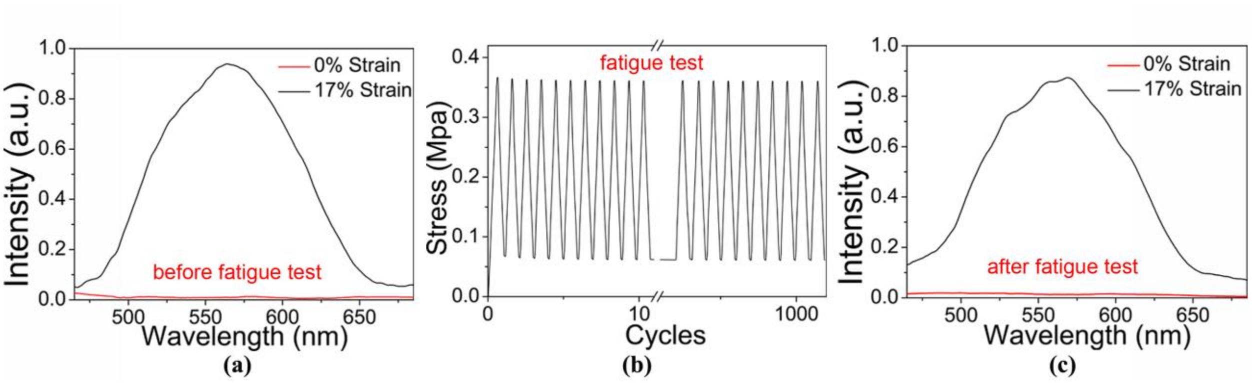

Supplementary Figure 4. (a) Fluorescent spectra of the EM at different strains as tested before the fatigue test (excitation wavelength of UV = 365 nm); (b) correlation between cyclic stress and cycles curve for the fatigue test; (c) fluorescence spectra of the EM at different strains as tested after the fatigue test (excitation wavelength of UV = 365 nm).

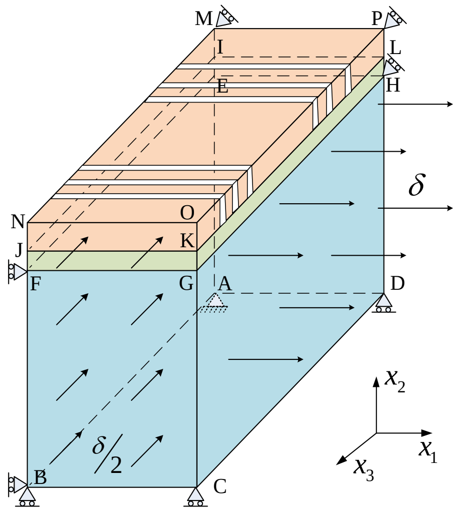

Supplementary Figure 5. Finite element model for the fold–ridge mechanism.

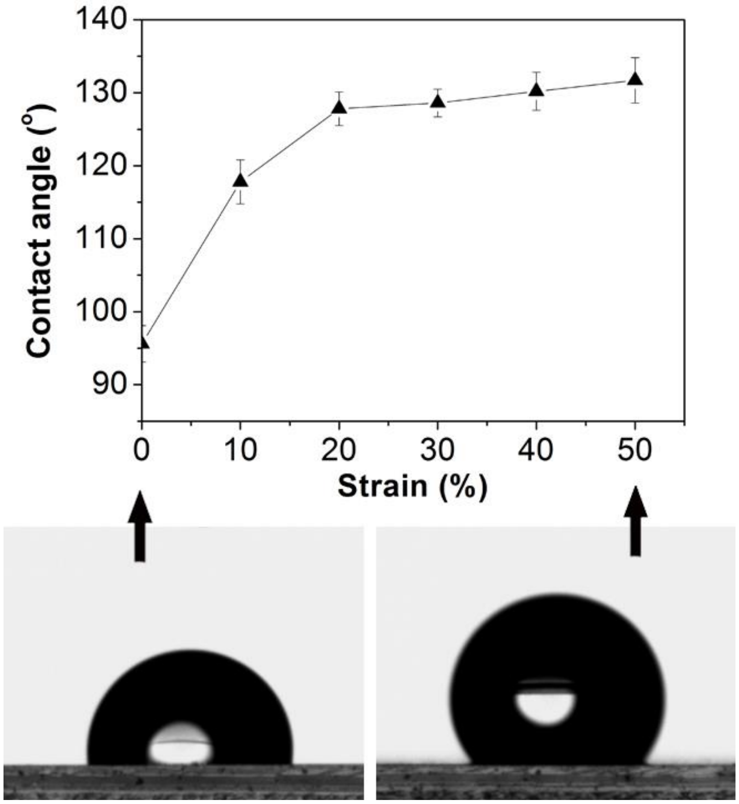

Supplementary Figure 6. Correlation of contact angle with applied strain on the topmost surface of transparency change mechanochromism. The sample was immersed in a 1 wt % glutaraldehyde solution for 1 h to form a PVA/laponite crosslinked network prior to test in order to achieve good water resistance[1].

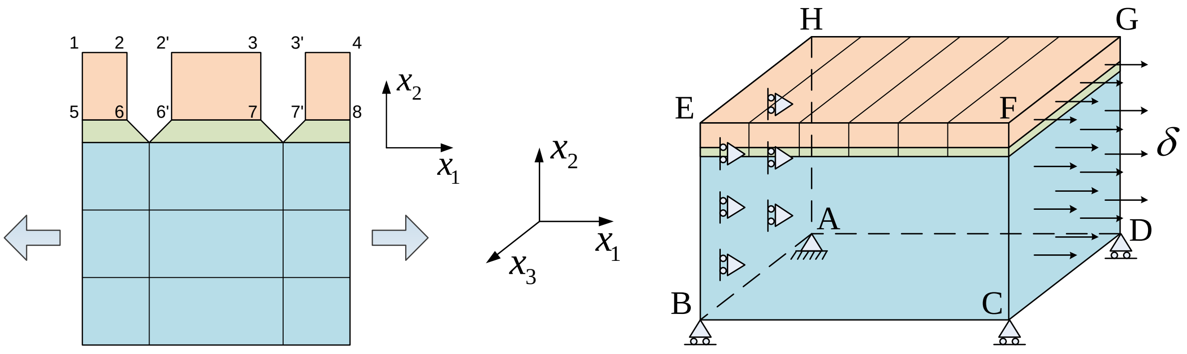

(a) (b)

Supplementary Figure 7. Finite element model for crack opening response. (a) Dummy nodes were used to represent crack opening. (b) Boundary conditions.

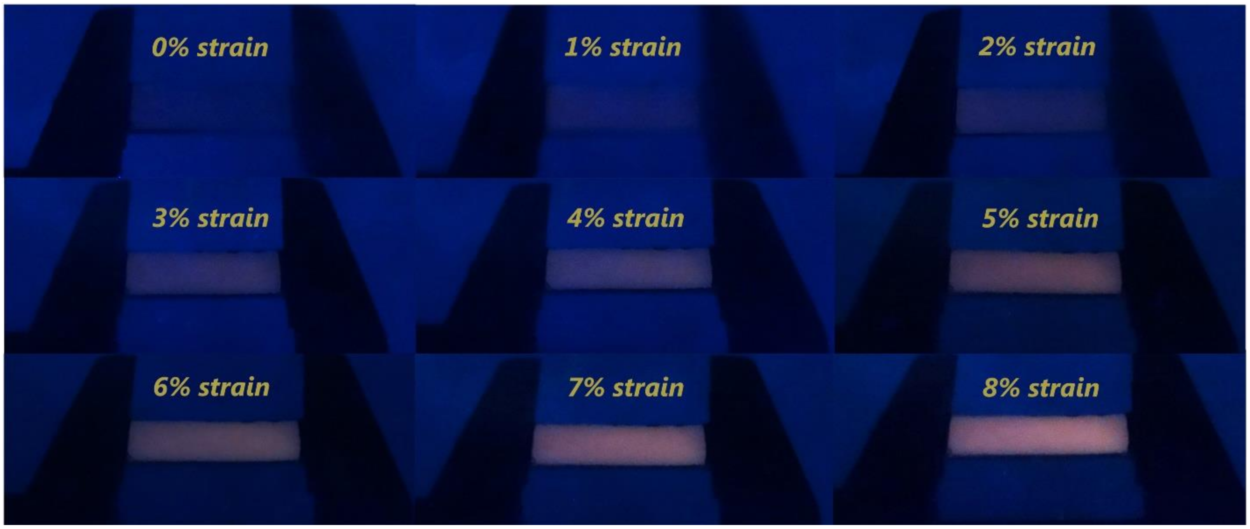

Supplementary Figure 8. Digital photos showing fluorescent intensity change as a functional of applied tensile stain (0-8%) in the luminescent mechanochromism (UV light source: λ=365 nm).

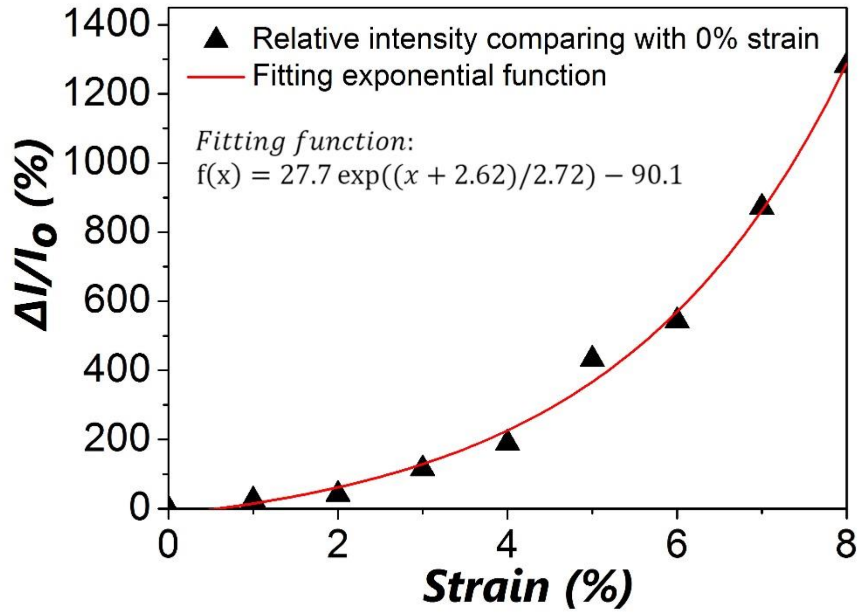

Supplementary Figure 9. Change of relative intensity ratio as a function of applied strain (0-8%) and the corresponding exponential function fitting in the luminescent mechanochromism.

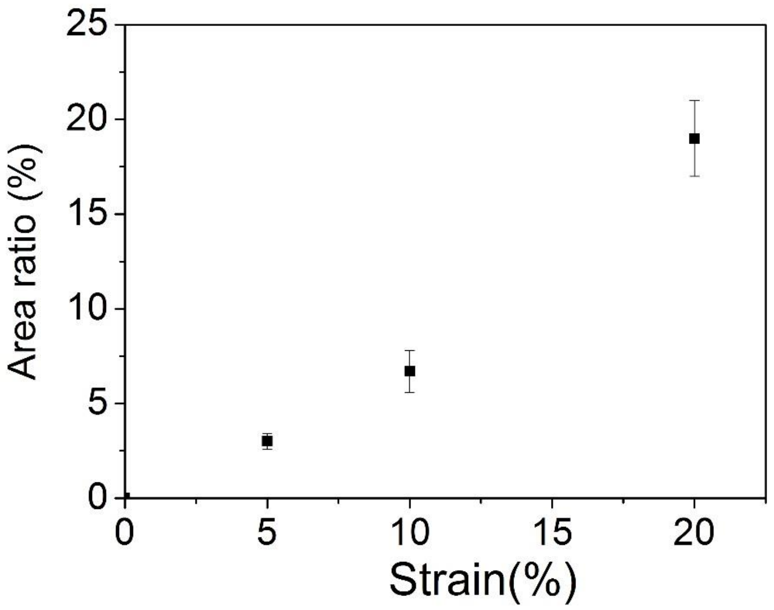

Supplementary Figure 10. The area ratio of opening crack to non-opening-crack surface in CAM.

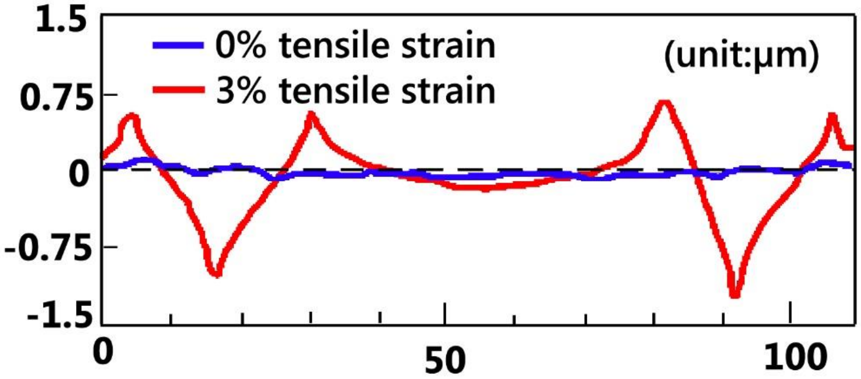

Supplementary Figure 11. The strain induced surface profile evolution of TCM on cross-section over X_1 direction as defined in Fig. 2. For the blue profile, the TCM experienced 0% tensile strain on X_1 direction and 0% compression strain on X_3 direction; for the red profile, the TCM experienced 3% tensile strain on X_1 direction and 1.5% compression strain on X_3 direction.

Supplementary Table

Supplementary Table 1. Thin film thickness and crack spacing used in the FE model.

	Film thickness (μm)	Crack spacing (μm)	Crack depth	
			Average (μm)	Standard deviation (μm)
LM	5.1	41	7.0	1.2
CAM	13.9	125	18.3	4.5

Supplementary Methods

Finite element simulation of the fold–ridge formation in TCM: When the film–substrate system is subjected to the longitudinal tension, the bilayer material undergoes compression in the transverse direction due to the Poisson's effect, resulting in the formation of folds and ridges as evident in the experiment (see Fig. 1 (d) and (e)). The computational model for the folding mechanism is similar to that for the crack evolution as shown in Supplementary Fig. 5. The rigid thin film was tied onto the PDMS substrate by enforcing the displacement continuity across the interface. The unit cell contains the material of one crack spacing in the longitudinal tension direction and two folds in the transverse compression direction. The film-substrate system was subjected to a state of biaxial loading that a uniform tension was prescribed on the PDMS substrate in the X_1 direction, while a uniform compression was applied on both the film and substrate in the X_3 direction, as shown in Fig. 2 (a). Since both the film and the substrate are incompressible, the magnitude of the transverse compressive strain (ε_3) was assumed to be half of the longitudinal tensile strain (ε_1).

The key to capture the reversible fold–ridge formation is to introduce damage in the thin film. During the pre-stretch stage, the development of invaginated folds and sharp ridges caused damage spots at the edges and the valleys. As a result, the material at these locations was treated as a damaging solid by reducing the modulus to 1% of the modulus of the pristine thin film. The evolution of the deformed shape is shown in Fig. 2 (a) and (c)-(f).

Finite element simulation of crack evolution in LM and CAM: The crack opening response was simulated using the commercial software ABAQUS (version 6.14). The PDMS substrate was modeled as an incompressible hyperelastic material using the Arruda-Boyce model with a ground state shear modulus of 0.32 MPa and a locking parameter (λ_m) of 1.17 [2]. The rigid thin film, modeled as an incompressible elastic solid with a Young's modulus of 10 GPa, was tied onto the substrate by enforcing the displacement continuity at the interface. Both the PDMS substrate and the thin film were meshed using 3D hybrid linear elements, C3D8H. The distributed cracks on the thin film, which were developed after the pre-stretch procedure, were modeled as dummy nodes at the crack interface. These cracks were fully opened through the thin film and arrested in the PDMS substrate, as schematically shown in Supplementary Fig. 7(a). In the present model, 10 parallel cracks were embedded along the loading direction, and the crack depth followed a normal distribution based on the average value and standard deviation measured from the experiment. The crack size during the deformation was determined by averaging the relative displacements of the two dummy nodes at the crack interface, d (see Supplementary Fig. 7(a)), of the 10 cracks.

The boundary conditions for the film–substrate system subjected to uniaxial tension are shown in Supplementary Fig. 7(b). A uniform displacement field is prescribed on the surface CDGF ($U_1 = \delta$), while the opposite surface (surface BAHE) is constrained along the x_1-direction ($U_1 = 0$). Additionally, point A is fixed ($U_1 = U_2 = U_3 = 0$) to prevent rigid body motion, and point B is restrained from moving along the x_2-direction ($U_2 = 0$). The film thickness, crack spacing, and crack depth used in the luminescent and color alteration mechanochromisms are summarized in Supplementary Table 1. In each case, a film-to-substrate

thickness ratio of 100:1 was maintained such that the PDMS substrate can be considered as an infinite medium.

Supplementary References:

1 Podsiadlo, P. *et al*. Ultrastrong and Stiff Layered Polymer Nanocomposites. *Science* **318**, 80-83 (2007).

2 Zang, J., Zhao, X., Cao, Y. & Hutchinson, J. W. Localized ridge wrinkling of stiff films on compliant substrates. *J. Mech. Phys. Solids* **60**, 1265-1279 (2012).
